# Brief Expositions of Practical Medicine, to Which Is Prefixed the Paradise of Doctors—a Fable

**Published:** 1859-05

**Authors:** 


ART. IV.—*Brief Expositions of Practical Medicine, to which is prefixed The Paradise of Doctors—a Fable.* By JACOB BIGELOW, M.D., late President of the Massachusetts Medical Society, Physician to the Massachusetts General Hospital, etc. Boston, 1858. pp. 69. 18mo.

IF brevity were the soul of philosophy, as it is represented to be of wit, there would be cause for gratulation at the sight of Dr. Bigelow's "Brief Expositions." But as both philosophy and wit consist of other ingredients besides brevity, we are required to look into the tiny volume before us, and ascertain whether these ingredients are present. Sometimes we hear of the whole of a disputed question being contained in a nut-shell; but what if we find the kernel to be withered or unsound? To what extent our search in the present case will be rewarded, or a pleasant flavored and nutritious kernel met with, will appear in the sequel. We turn with some curiosity to what is called "A Fable," and find it to be, not a symbolical representation, but a fictitious narrative of men's doings; not a fable so much as a parable; not characters drawn from the brute creation, the materials of which the fabulist makes use, but human actions set forth in a style of exaggeration. A real fable must have unity, and not be wanting in dignity, nor should it be without traces of gayety or satire.

Dr. Bigelow's fable, called "The Paradise of Doctors," is no fable, and therefore we need not look for the qualities of one in his production. We meet with exaggeration, but it wants generally even the humor of caricature. The paragraph beginning with "Clergymen and moralists forgot that men were sinful; it was quite enough that they were bilious," is a pleasant exception. If extremes in medical dogmas are deemed fit subjects for satire, it would be better to draw them from the history of medicine than from the annals of quackery, and certainly not to mix names illustrious in the former with the unprincipled adventurers in the latter. Why should "the heroic shades of Rush and Bouillaud" be supposed to be evolved by spirit-rappers in the same breath with those, we will not say of "Sangrado," but of Morrison and Brandreth? "The physic-taking part" of seamen's duty is not that of which, as a class, they can complain much. The requirements of their officers are too urgent and exacting to allow of the surgeon on board putting or keeping unnecessarily the men on the sick-list, without strong necessity. We have known two crews to pass each nearly six months at sea without their suffering at all from the contents of the medicine-chest or from the doctor, and a young one, too, who was with them all the time. "The spark which had struck the magazine caused the whole population to explode," is a very figurative sentence. The idea of a population, instead of a magazine exploding, is really a novel one; it goes beyond anything in this line to be found in Rabelais, and sinks the retail trade of the "young spoetizator" of that writer into insignificance.

The sudden and general awakening of "the physicians, druggists, and citizens of the quiet old State of Massachusetts," in regard to "the important duty of taking physic;" the universal doctoring and dosing, and manufacturing, and swallowing of drugs which followed, and the re-awakening to the consequences of this course, constitute the staple of the story of the "Paradise of Doctors," which, owing to the alarming influx of these gentlemen from the interior, and especially from New Hampshire, "became an excellent place for doctors to starve in." Among the interlocutors in the piece are three persons—an old lady, a bluff marketer, and a manufacturer, who give their experience, respectively, of homœopathy. An old gentleman gets up and delivers himself of his views on *rational medicine*, and the folly of extremes. He states:—

"That in the form of calomel, the City of New Orleans has swallowed up some hundreds of tons of the quicksilver of Spain and South America. Palaces were being built in various cities, alike from poisonous arsenic and harmless sarsaparilla. A century hence, the mines of gold will be sought for, not in California, but in the cemeteries of old cities, where it has been geologically deposited under the industry of dentists."

They who confound the fabulous with the fictitious may, on reading passages like this, think that, after all, our author has really written "a fable." The oracular old gentleman gravely expresses his belief—

"That medicine would have fulfilled its true mission when doctors should have enlightened the public on the important fact that there are certain things which medicine can do, and certain other things which it cannot do; instead of assuming for it the power to do impossibilities. Among the good effects which must ensue from the diffusion of light, would be the disappearance of quackery from the world; for quackery consists wholly in medication."

To this we would reply, most grave pundit, that, although such a line ought to be drawn for the sake of science, yet its most obvious effect would be to give up the division of incurable diseases to the deceptions and rapacity of quacks, whose strongest assertions of their superior skill are coupled with their alleged ability to cure these diseases. One little matter has escaped thee, O pundit! and that is, the inextinguishable desire of people of all ages and colors and creeds to prolong their lives, and to get rid of their diseases, and, as a corollary, to hope on, hope ever, in the belief that there are medicaments known to certain persons which will cure, ay, even that which others tell them is incurable. So soon as the regular physician assures his patient that his case is beyond the reach of medicine, said patient understands by this that his own doctor cannot cure him, and straightway the exit of the regular is a signal for the entrance of the irregular follower of Esculapius. The question here is not so much a better logic among medical men, although that is very desirable and much needed, but more enlightenment among the multitude, respecting the human frame, the functions of its several parts, and the means by which it is impressed and sustained by hygienic agents. Until a knowledge of this kind becomes general, we must deprecate any appeal to those outside of the profession; whether it be in the fictitious form described in the "Paradise of Doctors," or in the more customary fashion of quack advertisements.

When Dr. Bigelow's "Fable" and "Expositions" come before him for revision, we would suggest that he introduce, after his mentioning the quantity of quicksilver used by the people of New Orleans in the form of calomel, the case of the gigantic and renowned Pantagruel, the hero of Rabelais. It would be in keeping with the satire on over-drugging, although it may furnish, at the same time, a hint to some ingenious pill-vender to try still further public credulity. The treatment is narrated as follows:—

"Now, to tell you after what manner he was cured of his principal disease, I let pass; how, for a minorative, he took four hundred pound weight of colophonic scammony; sixscore and eighteen cart-loads of cassia; eleven thousand and nine hundred pounds weight of rhubarb; besides other confused jumblings of sundry drugs. You must understand that, by the advice of the physician, it was ordered that what did offend his stomach should be taken away; and, therefore, they made seventeen great balls of copper, each whereof was bigger than that which is to be seen at the top of St. Peter's needle, at Rome; and in such sort that they did open in the midst and shut with a spring. Into one of them entered one of his men, carrying a lantern and a torch lighted, and so Pantagruel swallowed him down like a little pill; into seven others went seven country fellows, having every one of them a shovel on his neck; into nine others entered nine wood-carriers, having each of them a basket hung to his neck; and so were they swallowed down like pills. When they were in his stomach, every one undid his spring and came out of their cabins, the first whereof was he that carried the lantern; and so they fell more than half a league into a most horrible gulf, more stinking and infectious than ever was Mephitis, or the marshes of Camerina, or the abominably and unsavory Lake of Serbina, whereof Strabo maketh mention. And had it not been that they had very well antidoted their stomach, heart, and wine-pot, which is called the noddle, they had been altogether suffocated and choked with these detestable vapors. * * * * * * * * * Finally they found a montjoy, or heap of ordure and filth: then fell the pioneers to work to dig it up, and the rest with their shovels filled the baskets, and when all was cleared away, every one retired himself into the ball. This done, Pantagruel, enforcing himself to a vomit, very easily brought them out ——; but when they came merrily out of their pills, I thought upon the Grecians coming out of the Trojan horse. By this means was he healed and brought into his former state and convalescence. And of those brazen pills you have one at Orleans, upon the temple of the body cross church."

The reader cannot fail to admire the conscientiousness of Rabelais, who, fearful that his narrative might be doubted, clinches it with an appeal to a visible and tangible evidence.

It is not necessary for us to follow Dr. Bigelow in his "Brief Expositions of Rational Medicine." Putting aside commonplaces and exaggerations, little remains in the shape of a demonstration of the real obstructions in the way of scientific progress in medicine, or of the principles to serve as a foundation for reform. The author talks of the evils of ultra-medication, polypharmacy, and conflicting modes of treatment. No person will contest with him on these points: they are universally admitted by medical men, who will also agree with the author, that a sick man ought to be saved from the officious good-will of his friends; and that officious medication ought to be prevented. But the author will not be echoed in his sweeping and indiscriminate denunciations of certain modes of practice,—as, for instance: "When carried to its 'heroic' extent, artificial medicine undermined the strength, elicited new morbid manifestations, and left more diseases than it took away." The common error consists in generalizing the heroic treatment; an error which would find its counterpart in a determined abstinence from such a treatment under all circumstances. A little farther on, under the head of "Rational Medicine," we find, however, the following acknowledgment:—

"The power of the medical art to palliate diseases is shown in a multitude of ways, active, cautious, and expectant. The pain of acute pleurisy is relieved by venesection; that of pleurodynia by anodynes and external applications. The pain of acute rheumatism is postponed by opium; that of gout by colchicum. Synovitis is favorably affected by rest; chronic rheumatism more frequently by exercise. Demulcents, opiates, and even astringents have their use in various irritations of the mucous membranes. Cathartics, laxatives, emetics, leeches, counter-irritants, cupping, hot and cold applications, etc., are of benefit in various local and general maladies."

A pretty good list this for a rationalistic doctor. The "heroical" practitioner would hardly ask for more, especially if he were permitted to fill up the terminal *et cetera* in his own fashion. We offer no criticism at the moment, on the limited pathology which looks to pain as the chief element in pleurisy, acute rheumatism and gout; or to the restricted view of the therapeutical agents mentioned, which would seem to limit their effects to the removal or mitigation of this pain. Dr. Bigelow's rationalism in medicine reminds us of the definition once made of orthodoxy by a zealous believer, in conversation with his friend, who differed from him on some question of theology. Orthodoxy, said the former, is my doxy,—heterodoxy is your doxy. Every physician will agree with the author in the superiority of rational medicine over all other methods of treating disease; but when the question comes up for consideration: What is the extent, what are the actual resources of rational or orthodox medicine? all the vexed questions on pathology and therapeutics, which have agitated the schools and tasked the powers of observation and reasoning of medical minds, from the earliest times down to the present day, are again opened. The author, in another page, would dismiss them all from court, as unworthy of argument and adjudication, by asserting, as his sincere belief, notwithstanding his previous admissions, as quoted above—

"That the unbiased opinion of most medical men of sound judgment and long experience is made up, that the amount of death and disaster in the world would be less, if all disease were left to itself, than it now is under the multiform, reckless and contradictory modes of practice, good and bad, with which practitioners of adverse denominations carry on their differences at the expense of their patients."

We deny that any such opinion is held by enlightened medical men, while, at the same time, we protest against the unjust manner in which the concluding part of the assertion is stated, as if the question of modes of treatment were a scholastic one, an intellectual contest merely, between doctors, in which patients had a secondary and minor interest, and were merely used as subjects for experiment, in place of being regarded, as they actually are, the principals, for the preservation of whose health, and for the cure of whose diseases, doctors strain their faculties to the utmost. The assertion itself is not susceptible of direct proof, and must be taken as the creed of an individual, certainly not of the thinking part of the profession; nor, on the other hand, can it be refuted by direct evidence. But there is a large amount of collateral proof which would go to nullify it; as, for instance, the general fact, that, with the increase of population and the advance of civilization, from the dark ages onward, there has been a proportionate extension of medical teaching and increase of medical men. It is also on record, that, during the last three centuries, the average duration of human life has been progressively increasing; and it is also equally clear, that medical schools have multiplied, medical doctrines have been more numerous, and modes of practice more diversified, during this period, than for many centuries before. M. Marc d'Espine, in a late work on Comparative Mortuary Statistics, shows that the *probable life* at Geneva in the sixteenth century was rather less than five years; in the seventeenth century, eleven years; at the beginning of the eighteenth, twenty-seven years; at the end of the century, thirty-two years; and now it is forty-four years. M. Mallet had previously shown, that the mean duration of life in Geneva in 1833, was nearly double (or as forty years five months to twenty-one years and two months) that reached more than two centuries before.* At the present time the probable life in Bavaria is still as low as from ten to twelve years; and yet, surprising as it must seem to Dr. Bigelow and the non-medical intervention school, three-fifths of those who die in that kingdom receive no medical attentions whatever; whereas in Geneva, the want of medical attentions to the sick is of rare occurrence. One would hardly expect to see, after Dr. Bigelow's dogmatic assertion of the injurious consequences of medical treatment, the large qualifications, in fact the contradiction in the sentences which immediately follow it, and which read thus:—

"But there is no probability that expectant medicine will ever prevail in its character as such. The amount of positive good which, in fifty centuries, art has brought to the assistance of medicine, although far more limited than we could desire, is, nevertheless, both sufficient and worthy to employ the talents of the best and most enlightened physicians."

We had been previously told by the author—

"The great objects which medical practice professes to effect, and which there can be no doubt that it frequently does effect, are the following: 1. The cure of certain diseases. 2. The relief and palliation of all diseases. 3. The

---

* Bell's *Regimen and Longevity*, pp. 23, 24.

safe conduct of the sick. In all these objects it sometimes fails; yet instances of its success are sufficiently numerous to establish the necessity of the existence of medicine as a profession."

We glean from the scattered propositions, postulates, and queer aphorisms of Dr. Bigelow, that medicine, as he understands it, is, after all, admissible, and may even do some good, provided it be practiced by physicians who are imbued with his notions and who adopt his creed. One's gravity is somewhat tasked by the following:—

"Before commencing any course of treatment in a given case, two questions should always be asked: 1. Will it do good? 2. Will it do harm? A right answer to these questions will not fail to produce a right practice."

Just so. When the physician can anticipate, on approaching his patient, the whole march, the complications and the terminations of the disease, and the effects of different remedies on it, he will be sure to know how to manage it. Some simple-minded men, on recovering from the surprise into which they were thrown by this Delphic announcement, will ask whether this does not suppose an entire solution of the problem, without our yet knowing the steps by which it can be reached, or seeing a single difficulty that stood in the way removed. We confess to be among the number of the simple-minded. As far as we can get at the meaning of the author, his statements of medical errors and his recommendations of medical reform, though vaguely and contradictorily set forth, amount to the inculcation of a closer adherence to medical logic than obtains among either teachers or practitioners of medicine. Would that the excellent volume of Oesterlen were made a text-book in our schools—as a guide to a better and improved system of medical observation and of medical reasoning.

There is reason to believe that neither the "Rational Expositions" of Dr. Bigelow, nor the kindred work of Sir John Forbes, "Nature and Art in the Cure of Disease," can stand the scrutiny of medical logic, on the very point which the authors may be supposed to regard, in their reformatory labors, with the most complacency. Our reference is "Nature," as in their eyes the great curer and restorer. What, we would ask, is nature? We gain nothing in positive medicine by being told that all our medical agents are often of no avail, or are mischievous in the treatment of disease, and that our reliance ought to be on nature. But we repeat the question: What or who is nature? We find her personified, a *Bona Dea,* who watches over all the processes of the vital organism, rectifying them where they have gone wrong,—amending where the material has become damaged and imperfect,—casting out old and effete, and bringing in new and plastic substances. Is not this a mere play of imagination, which conveys no instruction, points to no ascertainable way, suggests no measurable means of cure? What is said by A. Comte of all knowledge, applies to medicine, viz., that it has to run through three stages: 1. That of fiction and poetry, simple faith, and dogmatic opinion. 2. Of arbitrary hypotheses, *a priori* abstraction, combination, or speculation. 3. The stage of sober but positive science, based upon a clear understanding of what it has to deal with.* Medicine is in the second of these stages, and is advancing to the third one; but the modern worshipers of Nature would, if their notions were adopted, throw it back into the first stage. Having erected nature into a kind of providence—and indeed it would be just as scientific and as explanatory to speak of the Deity himself setting up healing and curative and restorative processes as Nature doing these things—we suppose her to go to work with such a design, and according to such laws and principles as we just happen to be acquainted with. This leads to mysticism, and replaces the superstition of former times, when diseases were attributed to diabolic agency, and cures were believed to be performed by witchcraft. Disease is now often regarded as an existing thing and is to be driven away by Nature, another existing thing, and thus we have a double ontology. Nor is the substitution of vital force any better or more scientific. Little is gained in true or useful knowledge of medicine, by assuming that nature or the vital force bring about curative efforts, favorable crises, and finally a cure. But we have not room to pursue the subject, and must content ourselves with this brief glimpse of some of its bearings.

* F. Oesterlen, Medical Logic, p. 427.

